# Methodological Assessment of PDMS Passive Sampling for Skin VOC Collection Across Body Sites

**DOI:** 10.3390/metabo16100728

**Published:** 2026-09-29

**Authors:** Seibi Kobara, Min Huang, Rizky Ilhamsyah, Daniel Struk, John Dimandja, Peter J. Hesketh, Daniela Chanci Arrubla, Chase Fensore, Carmen C. Polito, Rishikesan Kamaleswaran, Annette Esper

**Affiliations:** 1 Department of Computer Science and Informatics, Emory University, 400 Dowman Drive, Atlanta, GA 30322, USA; min.huang@duke.edu (M.H.); chase.fensore@emory.edu (C.F.); 2 Department of Biomedical Informatics, Emory University School of Medicine, 100 Woodruff Circle, Atlanta, GA 30322, USA; 3 Department of Surgery, Duke University School of Medicine, 2301 Erwin Rd, Durham, NC 27707, USA; daniela.chanciarrubla@duke.edu; 4 Department of Anesthesiology, Duke University School of Medicine, DUMC 3094, Durham, NC 27710, USA; 5 George W. Woodruff School of Mechanical Engineering, Georgia Institute of Technology, 801 Ferst Dr, Atlanta, GA 30332, USA; r_ilhamsyah@gatech.edu (R.I.); struk.daniel@gmail.com (D.S.); jdimandja3@gatech.edu (J.D.); peter.hesketh@me.gatech.edu (P.J.H.); 6 Department of Biomedical Engineering, Duke University Pratt School of Engineering, 1427 Fitzpatrick Center, Box 90281, Durham, NC 27708, USA; 7 Division of Pulmonary, Allergy and Critical Care Medicine, Emory University School of Medicine, 100 Woodruff Circle, Atlanta, GA 30322, USA; cpolito@emory.edu (C.C.P.); aesper@emory.edu (A.E.); 8 Department of Electrical and Computer Engineering, Duke University Pratt School of Engineering, 130 Hudson Hall Box 90291, Durham, NC 27708, USA

**Keywords:** volatile organic compounds, pre-clinical, polydimethylsiloxane, skin, gas chromatography–mass spectrometry

## Abstract

**Highlights:**

**What are the main findings?**

- Skin-contact PDMS detected 160 putatively annotated VOCs using a simplified sampling procedure in four healthy volunteers.
- Substantial VOC overlap was observed across skin-contact, clothing, room, and blank PDMS conditions.

**What are the implications of the main findings?**

- Simplified skin-contact PDMS is feasible for capturing diverse VOC signals and warrants validation in larger and clinically relevant cohorts.
- The observed overlap of VOCs across experimental conditions illustrates challenges that may arise when applying skin VOC sampling in clinical and real-world environments where strict environmental control may be infeasible.

**Abstract:**

**Background/Objectives:** Polydimethylsiloxane (PDMS) is a non-invasive and versatile material often used for non-invasive collection of skin-emitted volatile organic compounds (VOCs), with potential applicability in acute and pre-critical care settings. However, most existing PDMS-based methodologies rely on extensive sample preparation and environmental control, limiting their feasibility in time-sensitive clinical contexts. **Methods:** We conducted a proof-of-concept pilot case-series study in four healthy volunteers to evaluate whether a simplified skin-contact PDMS sampling procedure can capture detectable VOCs and preserve individual-level variation. PDMS strips were applied directly to the skin with minimal preparation, and collected VOCs were analyzed using gas chromatography–mass spectrometry. Donor-associated variability was assessed using Bray–Curtis dissimilarity, and variability in VOC detection was evaluated across body sites. **Results:** Skin-contact PDMS sampling detected 160 VOCs across four participants. The mean within-donor Bray–Curtis dissimilarity was 0.308, compared with a mean between-donor dissimilarity of 0.347. VOC detection variability differed across body sites, with lower coefficients of variation at the forehead, neck, and wrist than at the ankle. **Conclusions:** Under simplified sampling conditions, skin-contact PDMS captured individual-associated VOC profiles with lower within-donor variability than between-donor variability. These findings support the feasibility of PDMS-based skin VOC sampling in minimally controlled settings. Further validation in larger and clinically relevant cohorts is warranted to assess the utility of PDMS-sampled skin VOCs as potential biomarkers for early disease detection.

## 1. Introduction

Volatile organic compounds (VOCs) are a broad class of organic chemical compounds, characterized by high volatility at room temperature and molecular mobility [1,2]. VOCs are emitted through both natural and anthropogenic processes, including wildfires and industrial activities, and have been associated with immune activation and disease risk [2,3]. Accumulating evidence indicates that VOCs are also continuously emitted by humans through breath and skin, reflecting endogenous metabolism, microbial activity, and environmental exposure [4,5,6].

Several methodologies have been developed to collect skin-emitted VOCs, including polydimethylsiloxane (PDMS), solid-phase microextraction (SPME), cotton pads, Tenax/Carboxen sorbents, and glass beads [6]. These approaches are generally passive and non-invasive, making them well suited for studying VOC variation across individuals and environments. Nevertheless, substrates such as cotton and porous sorbents may suffer from high environmental signals, batch-to-batch variability, and limited chemical selectivity, which can compromise quantitative reliability [7]. PDMS-based skin-contact sampling offers several advantages, including a high partition coefficient for nonpolar and semi-volatile compounds, low chemical reactivity, and minimal endogenous contamination [8]. The ability to perform solvent-free thermal desorption further simplifies the analytical workflow and reduces analyte loss. In summary, these properties make PDMS an attractive platform for profiling skin-emitted VOCs in both clinical and real-world environments [9].

Critical illnesses such as sepsis and acute respiratory distress syndrome (ARDS) remain leading causes of global morbidity and mortality [10,11,12], with clinical downstream outcomes strongly influenced by the timeliness of detection and intervention [13]. Despite advances in critical care, early or mild-to-moderate ARDS is frequently underrecognized [11], highlighting the need for methods capable of capturing early physiological dysregulation during pre-critical or rapidly evolving states. In emergency or resuscitation settings, conventional sample collection (e.g., blood draws) may be infeasible or contraindicated, particularly in hemodynamically unstable patients. In this context, skin-contact VOC sampling using PDMS represents a potentially feasible alternative.

However, most existing skin VOC studies rely on extensive PDMS preparation protocols and tightly controlled environmental conditions [6,14,15,16], which enhance the specificity of VOC capture but limit logistical feasibility in pre-critical and critical settings. In these environments, physiologic changes during resuscitation may provide valuable insight into patient status and prognosis, yet conventional sampling approaches are often impractical. To address this gap, we propose a simplified PDMS-based skin-contact sampling procedure that can be applied and left in place, enabling passive, time-integrated collection of chemical signals during resuscitation. This “set-and-forget” approach captures cumulative exposure rather than a single time-point snapshot. However, the performance of such minimally controlled PDMS-based sampling—particularly with respect to VOC detectability, individual variability, and susceptibility to environmental and material-derived contributions—remains insufficiently characterized.

In this pilot case series, we evaluated whether a simplified PDMS-based skin-contact sampling procedure, requiring only minimal skin preparation, can capture VOCs consistent with previously reported human-associated compounds. We further assessed the extent to which clothing, room environment, and material-derived VOCs contribute to the observed signal using parallel control samples. This work is intended as a proof of concept to characterize VOCs detected in skin-contact PDMS and to inform future validation and methodological refinement, rather than to generate generalizable biological conclusions.

## 2. Materials and Methods

### 2.1. Study Setting and Participants

We conducted a pilot feasibility study involving skin VOC sampling from four healthy adult volunteers. The study was designed to evaluate the technical feasibility of VOC collection procedures and was descriptive in nature.

This work was not intended to generate generalizable knowledge and case series of five or fewer subjects. This research was reviewed by the Emory Institutional Review Board and determined to qualify as non-human subjects research. All procedures were conducted in accordance with the Declaration of Helsinki.

### 2.2. Skin VOC Collection Procedure

Polydimethylsiloxane (PDMS) tape (SSP-M823), an FDA-compliant membrane, was purchased from Specialty Silicone Products, Inc (New York, NY, USA) [17]. PDMS strips (3.0 mm × 15.0 mm) were laser-cut using a Trotec Speedy Laser 300 (Trotec Laser GmbH, Plymouth, MI, USA) with Ruby software (2024 release). Prior to sampling, PDMS strips were thermally conditioned to reduce background volatile contaminants at 356 °F for 60 min, allowed to cool to room temperature, and stored in a glass tube, isolated individually.

For proof-of-concept testing, PDMS strips were applied to four body sites: forehead, neck, wrist, and ankle (Figure 1 left). Before application, participants washed the forehead and hands with soap and paper-dried the skin. PDMS strips were placed directly onto the skin at each site, wrapped by aluminum foil, and secured with 3M Nextcare Bandage (Figure 1 right). In addition to skin-contact sampling, several PDMS control conditions were prepared, including PDMS attached to clothing on the right thigh (clothing control), PDMS left exposed in a chemical laboratory environment during the sampling period (room control), and unexposed blank PDMS (blank control). Participants then walked outdoors in a university environment for 50 min to allow passive absorption of skin-emitted and environmental VOCs.

After sampling, PDMS strips were removed using clean forceps, inserted into a glass inlet, and stored at −80 °C using dry ice until analysis. Collected compounds were analyzed by gas chromatography–mass spectrometry (GC-MS) using Agilent 6890 gas chromatography system (Agilent Technologies, Santa Clara, CA, USA) equipped with thermal desorption unit and coupled with a time-of-flight (TOF) mass spectrometer (LECO Pegasus 4D, St. Joseph, MI, USA). Chromatographic separation was performed on a 30 m Rxi-5 column (250 μm ID, 0.25 μm film thickness) using helium at 1 mL/min. Samples were introduced in splitless mode, and the oven temperatire was ramped from 40 °C to 250 °C at 10 °C/min. Mass spectra were acquired in electron ionization mode (70 eV) over a mass range of 30–250 amu at 200 spectra/sec. Detailed GC-MS conditions and quality control procedures are provided in the Supplementary Methods.

Identified VOCs were mapped to InChIKey identifiers using CIRpy (version 1.0.2) [18], a Python interface to the Chemical Identifier Resolver (CIR) developed by the Computer-Aided Drug Design (CADD) Group at the National Cancer Institute (NCI/NIH). Chemical superclass, class, and subclass annotations were subsequently assigned using the classyfireR [19] package in R.

### 2.3. Statistical Analysis

Raw features were detected and aligned across all samples. Features were excluded if they were detected in fewer than four samples. Samples were further filtered if the number of detected compounds was less than 10% of the total number of qualified features retained after feature-level filtering.

To assess inter-individual (donor) and body-site variability, β diversity was evaluated using the Bray–Curtis dissimilarity metric. β diversity analyses were performed using compound abundance profiles restricted to VOCs detected using skin-contact PDMS.

To evaluate variability in compound detection performance across body sites, the proportion of detected compounds (defined as non-zero peak intensity) was compared among body sites.

We next compared VOC features detected using skin-contact PDMS with those detected using multiple control conditions, including clothing, room, and blank controls. In addition, we compared detected VOCs with a curated compilation of previously reported PDMS-derived healthy human-associated VOCs from Mitra et al. [6].

As a sensitivity analysis, we evaluated whether the observed patterns were robust to compound annotation quality. Features were restricted to those with both similarity and reverse similarity scores > 700 against the reference spectral library. Using this restricted feature set, donor- and body-site variability and the distribution of features across skin-contract PDMS and control conditions were re-evaluated.

Given the case-series design and limited sample size, the study was not designed to draw generalizable knowledge. Therefore, no formal inferential statistical comparisons were performed for inter-individual or body-site variability, and the reported coefficients of variation were used solely as descriptive measures. All preprocessing and statistical analyses were performed using R (version 4.4.1).

## 3. Results

### 3.1. Detected Compounds Using PDMS

PDMS-based skin VOC sampling was conducted as a proof-of-concept study with healthy adult donors. Four donors were sampled across four body sites (forehead, neck, wrist, and ankle), yielding a total of 13 PDMS samples for analysis; one donor did not undergo forehead and neck sampling for esthetic reasons, and one sample was removed based on call rate. Following feature detection, alignment, and quality-control filtering, 160 putatively annotated VOCs were retained for downstream analyses. Given a limited sample size and the scope of a pilot case series, subsequent analyses focused on describing the variability of VOC collection across donors and body sites.

### 3.2. Donor and Body-Site Variability

Skin-contact PDMS sampling yielded 160 putatively annotated VOCs, including compounds such as acetone, octanal, and decanal (Supplementary Materials Table S1). These putatively annotated VOCs were used to assess donor- and body-site-specific variability. Bray–Curtis dissimilarity was computed across all samples and visualized using principal coordinate analysis (Figure 2). The mean within-donor dissimilarity was 0.308, compared with a mean between-donor dissimilarity of 0.347.

To evaluate the consistency of VOC detection across body sites, the compound abundance matrix was binarized based on the presence or absence of a detectable signal (non-zero peak intensity). The proportion of detected compounds was calculated for each body site. The coefficient of variation (CV) was lowest for the forehead (CV = 0.043), followed by the neck (CV = 0.088), wrist (CV = 0.105), and ankle (CV = 0.615) (Figure 3).

### 3.3. VOC Overlap Across Skin-Contact PDMS and Control PDMS Samples

We next compared VOCs detected from skin-contact PDMS with VOC profiles obtained from multiple control conditions, including clothing, room, and blank controls. All control samples were taken under the same conditions at the same time and location as the skin sample collection, to provide relevant sample reference for comparison with the skin-based sampling. In addition, we compared detected VOCs with a curated compilation of previously reported PDMS-derived healthy human-associated VOCs from Mitra et al. [6]. This is a pilot study and shows the feasibility of this method.

Among VOCs detected using skin-contact PDMS, 94 putatively annotated VOCs were also detected in clothing-control samples, 99 putatively annotated VOCs were detected in room-control samples, and 29 putatively annotated VOCs were detected in blank-control samples (Figure 4). VOCs overlapping across skin-contact PDMS, clothing controls, room controls, and blank controls included benzothiazoles, aldehydes (e.g., octanal, nonanal, decanal), and fatty acids (e.g., n-decanoic acid). VOCs detected in intersections across experimental conditions are provided in Supplementary Materials Table S1.

We further compared VOCs detected in skin-contact PDMS with a collection of PDMS-derived healthy human-associated VOCs curated by Mitra et al. [6]. A total of 80 putatively annotated VOCs reported in that compilation were not detected in any experimental condition in our study. These undetected compounds included alcohols and polyols (e.g., dihydromyrcenol and ethanol), short- to medium-chain aldehydes (e.g., heptanal, (E)-2-nonenal, and 2-decenal), and short- to medium-chain ketons (2-Nonanone, 2-Decanone, and Cyclopentanone).

### 3.4. Sensitivity Analysis

To assess the robustness of the primary findings to spectral-library annotation quality, we repeated the analyses after restricting features to those with similarity and reverse similarity scores > 700. Among the 160 putatively annotated VOCs detected using skin-contact PDMS, 108 met these spectral-matching criteria. Using this restricted feature set, the mean within-donor Bray–Curtis dissimilarity was 0.277, compared with a mean between-donor dissimilarity of 0.304 (Supplementary Materials Figure S1A).

Body-site variability was also re-evaluated using the restricted feature set. The wrist showed the lowest coefficient of variation (CV = 0.060), followed by the forehead (CV = 0.078), neck (CV = 0.109), and ankle (CV = 0.594) (Supplementary Materials Figure S1B). Although the site with the lowest CV differed from the primary analysis, in which the forehead showed the lowest CV, the overall estimates remained descriptive given the small number of observations at each body site.

Substantial overlap between skin-contact PDMS and the control conditions was preserved after application of the spectral-matching criteria (Supplementary Materials Figure S1C and Supplementary Materials Table S2). Putatively annotated benzothiazoles and aldehydes, including octanal, nonanal, and decanal, remained shared across skin-contact PDMS, clothing, room, and blank controls. In comparison with the previously reported VOC compilation, 82 higher-confidence putatively annotated VOCs were not detected under any experimental condition in our study. These included alcohols and polyols (e.g., dihydromyrcenol and ethanol), short- to medium-chain aldehydes (e.g., heptanal, (E)-2-nonenal, and 2-decenal), and ketones (e.g., 2-nonanone, 2-decanone, and cyclopentanone). Overall, the major descriptive patterns were preserved after restricting the analysis based on spectral-matching quality.

## 4. Discussion

In this pilot study, we evaluated the feasibility of using PDMS for the detection of human-associated VOCs under simplified and practical sampling conditions, incorporating multiple quality control procedures. Among the 160 putatively annotated VOCs detected in the primary analysis, within-donor variability was lower than between-donor variability, suggesting that the PDMS-based approach may be a potential procedure to capture individual-level VOC profiles. Across body sites, the forehead, neck, and wrist exhibited the lowest coefficients of variation, indicating greater stability of VOC detection at these locations.

PDMS represents a non-invasive, low-burden sampling material with particular relevance for clinical and critical care settings [8,9], where rapid deployment and minimal infrastructure are essential. Although PDMS-based VOC sampling has been widely explored, most prior studies relied on extensive skin-cleaning protocols and tightly controlled environments [6], limiting real-world applicability. In contrast, we evaluated a simplified preparation procedure and explicitly assessed VOC profiles across multiple control conditions. Despite this minimal preparation, the observed donor-specific signal—reflected by significantly lower within-donor than between-donor variability—supports the capacity of PDMS sampling to capture meaningful individual differences in VOC profiles.

Body site was associated with differences in the observed variability of VOC detection. While the forehead demonstrated the lowest variability, practical considerations such as cosmetics or topical products may limit its routine use. In contrast, the wrist may represent an accessible alternative for PDMS-based VOC sampling in applied or clinical contexts. However, larger studies with repeated sampling are needed to evaluate reproducibility and determine the optimal body site for the simplified skin-contact PDMS procedure.

Comparison across experimental conditions indicate that skin-contact PDMS captures a composite VOC signal influenced by skin-associated chemistry as well as the surrounding microenvironment. Notably, benzothiazoles were detected across experimental conditions, indicating contributions from non-biological sources to skin-associated VOCs [20,21]. While blank controls remain useful for identifying process-related artifacts, the substantial overlap was observed across skin-contact PDMS, clothing controls, room controls, and blank controls, including aldehydes and ketones. Although the direct origin of these compounds cannot be definitively assigned, their presence across multiple conditions suggests complex transfer dynamics, including skin-to-PDMS absorption, ambient air-to-skin deposition, accumulation on clothing, and indirect exchange between these reservoirs [22]. These findings indicate that strict exclusion of overlapping compounds may not be optimal for real-world VOC sampling.

When compared with a curated compilation of PDMS-derived healthy human-associated skin VOCs reported by Mitra et al. [6], 80 of 108 putatively annotated VOCs were not detected under any condition in our study. These undetected compounds included alcohols and polyol, medium-chain aldehydes, and short- to medium-chain ketons, which are volatile compounds. In contrast, our skin-contact PDMS sampling uniquely detected compounds such as pentadecanal and 1-hexadecanol, representing a long-chain aldehyde and fatty alcohol, respectively. These compounds are associated with lipid metabolism and oxidative stress [23,24], suggesting that the simplified, passive sampling approach may preferentially capture less volatile, accumulation-prone metabolites at the skin interface.

In future applications targeting acute or pre-critical care settings, individual variability and environmental heterogeneity are expected and largely uncontrollable. Laboratory handling conditions, available sanitizers, personal protective equipment, and emergent medical procedures may vary substantially in urgent clinical scenarios. In our experiment, substantial overlaps were observed between skin-contact PDMS and control settings. Rather than relying on exclusion or subtraction-based filtering of control-derived VOCs, future studies could investigate computation-based algorithm to account for dynamic transitions across skin-contact PDMS and other environmental controls. Such approaches may help distinguish physiological signals from environmental contributions; however, the present case series was not designed to evaluate or validate these methods. Future studies should first evaluate clinical workflow compatibility, inter-day reproducibility, storage stability, environmental confounding, and performance in relevant patient cohorts.

Several limitations warrant consideration. This study involved a small number of healthy volunteers and was not designed to evaluate biological or mechanistic inference across individuals. In addition, given the limited sample size, individual demographics and clinical characteristics were not incorporated into the analyses, limiting the interpretation of VOC profiles and potential inter-individual variability. Studies with larger sample sizes are needed to characterize inter-individual VOC variability and differences in VOC distributions across sampling conditions and to investigate their potential biological relevance.

Second, in this case-series study, skin-PDMS VOC profiles were compared across donors and different body sites. Given the small sample size, however, observed donor-level and body-site variability should be interpreted with caution. Additional biological samples will be necessary to determine whether the proposed simplified PDMS collection procedure and specific body sites can reliably capture individual-associated VOC patterns.

Third, we compared VOCs detected by skin-contact PDMS with those detected under three different control conditions, including clothing, room, and blank controls, while these control samples were taken under the same conditions at the same time and location as the skin sample collection. However, future applications may involve substantially more heterogeneous environments, where environmental confounding may be more pronounced and strict environmental control may not always be feasible. Within this simplified analytical design, substantial overlap in VOCs was observed across the skin-contact PDMS and control conditions, suggesting that exclusion of VOCs based on their control settings may also remove signals detected in skin-contact samples. Further studies with larger sample sizes are needed to characterize the dynamic transfer across clothing or ambient air and establish appropriate background-correction strategies.

Lastly, neck and forehead samples were missing from one participant due to esthetic concerns. Given the small sample size, these missing observations may substantially influence the descriptive estimates of body-site variability. The reason for missingness highlights practical considerations in body-site selection. In real-world applications, exposure areas such as the forehead and neck may be affected by participant preferences and the use of cosmetic or personal-care products, which could introduce additional exogeneous VOCs. Future studies should therefore consider both participant acceptability and potential behavior-related contamination when selecting standardized body sites for VOC collection.

## 5. Conclusions

In this pilot case series, we quantified VOCs captured by skin-contact PDMS using parallel clothing, room, and blank controls under a skin-cleaning-only preparation. VOC profiles obtained from skin-contact PDMS exhibited lower variability within individuals than between individuals, providing preliminary evidence that warrants future investigation of individual-level VOC patterns in larger studies. The substantial overlap of VOCs across skin, clothing, room, and blank controls highlights the importance of considering the complexity of VOC transfer and accumulation in real-world settings. Rather than strict exclusion of overlapping compounds in applied or acute care contexts, future studies may investigate computational frameworks that account for dynamic interactions between skin-derived VOCs and environmental or procedural influences across both short- and longer-term exposure timescales.

## Figures and Tables

**Figure 1:**
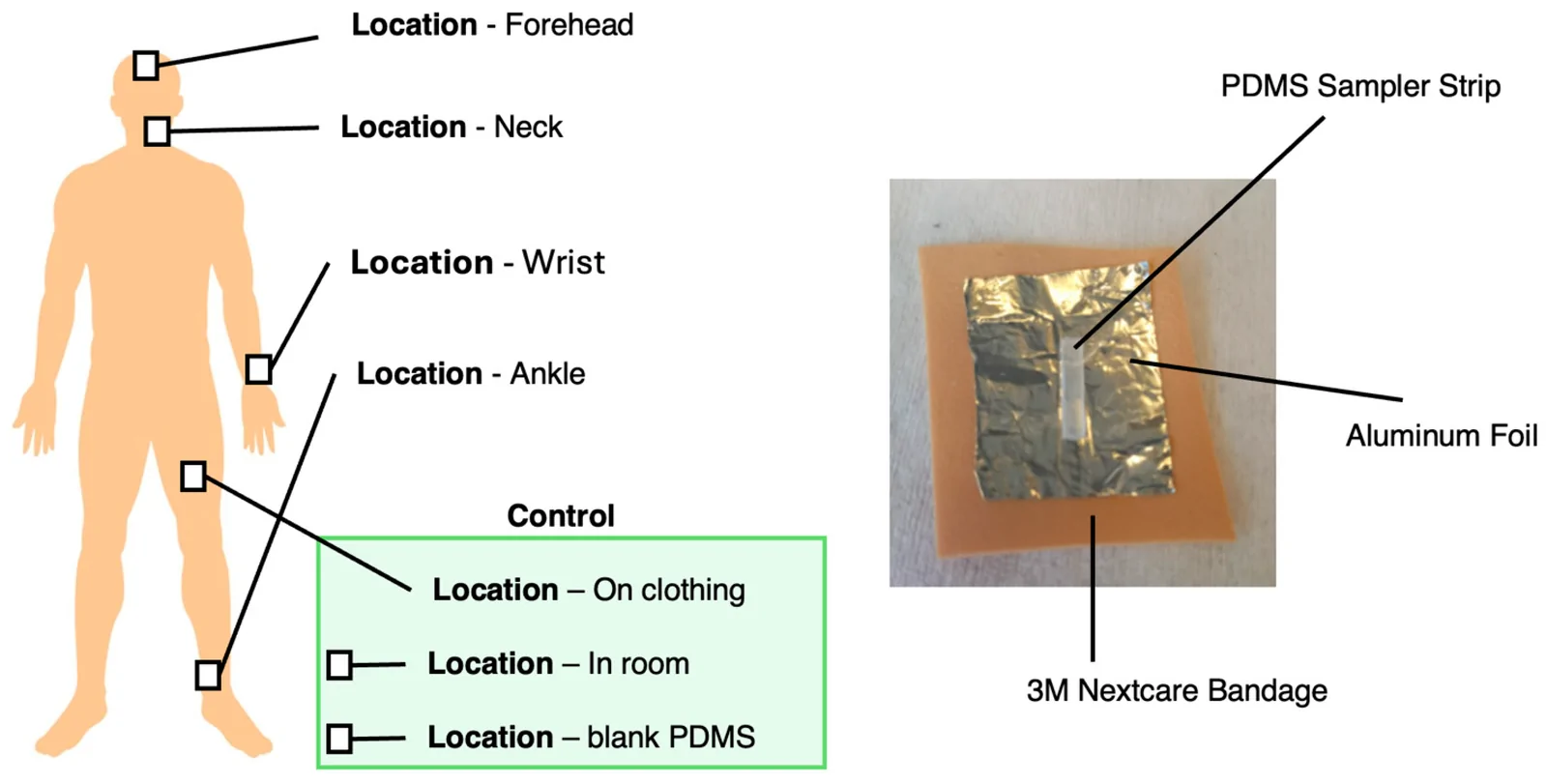
Overview of the experimental design, including the four skin sampling sites, three control conditions, and PDMS tape preparation procedure used for volatile organic compound (VOC) collection.

**Figure 2:**
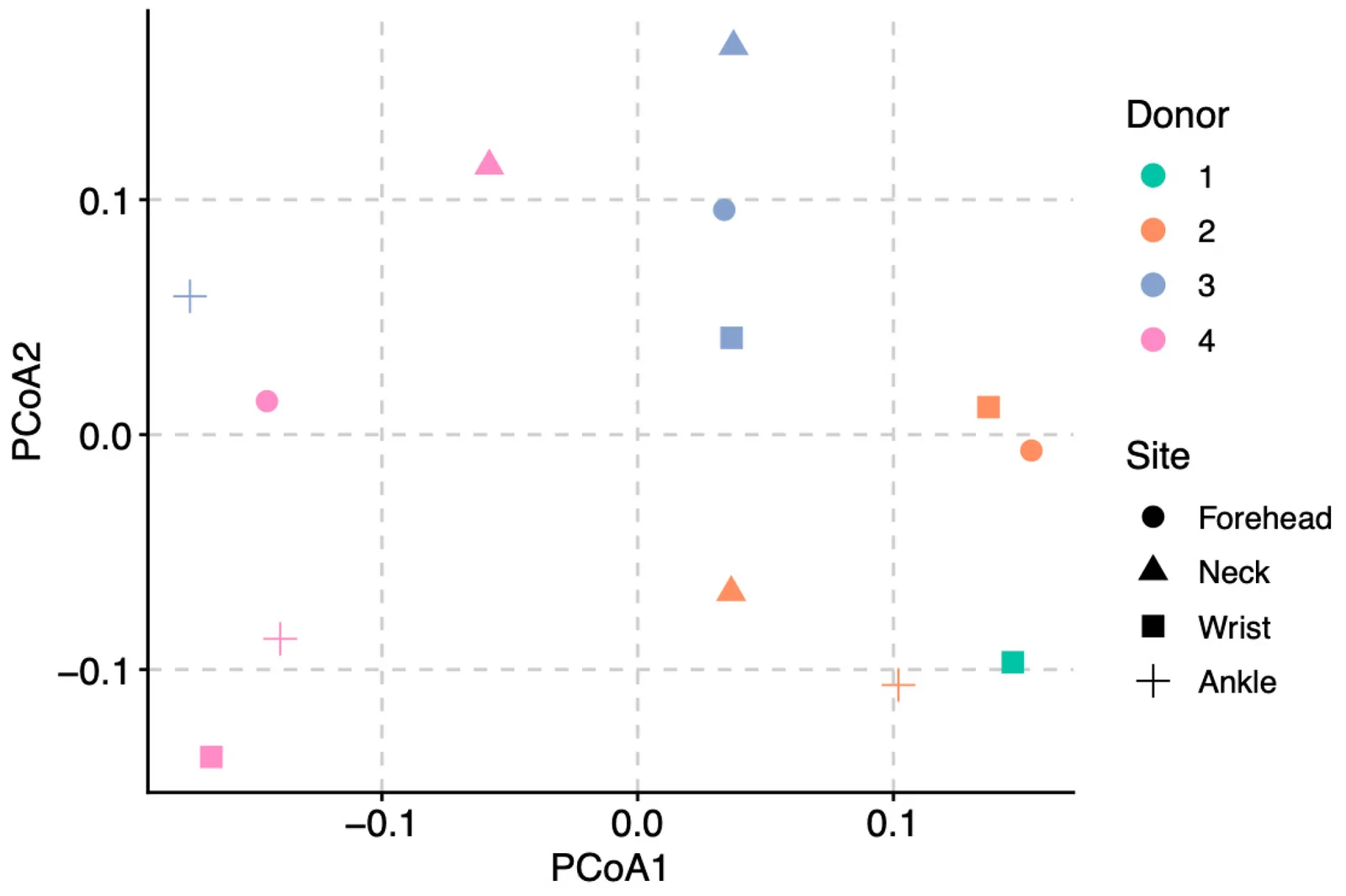
Principal coordinate analysis of Bray–Curtis dissimilarities across donor and body sites for the compound measurements across 160 detected VOCs.

**Figure 3:**
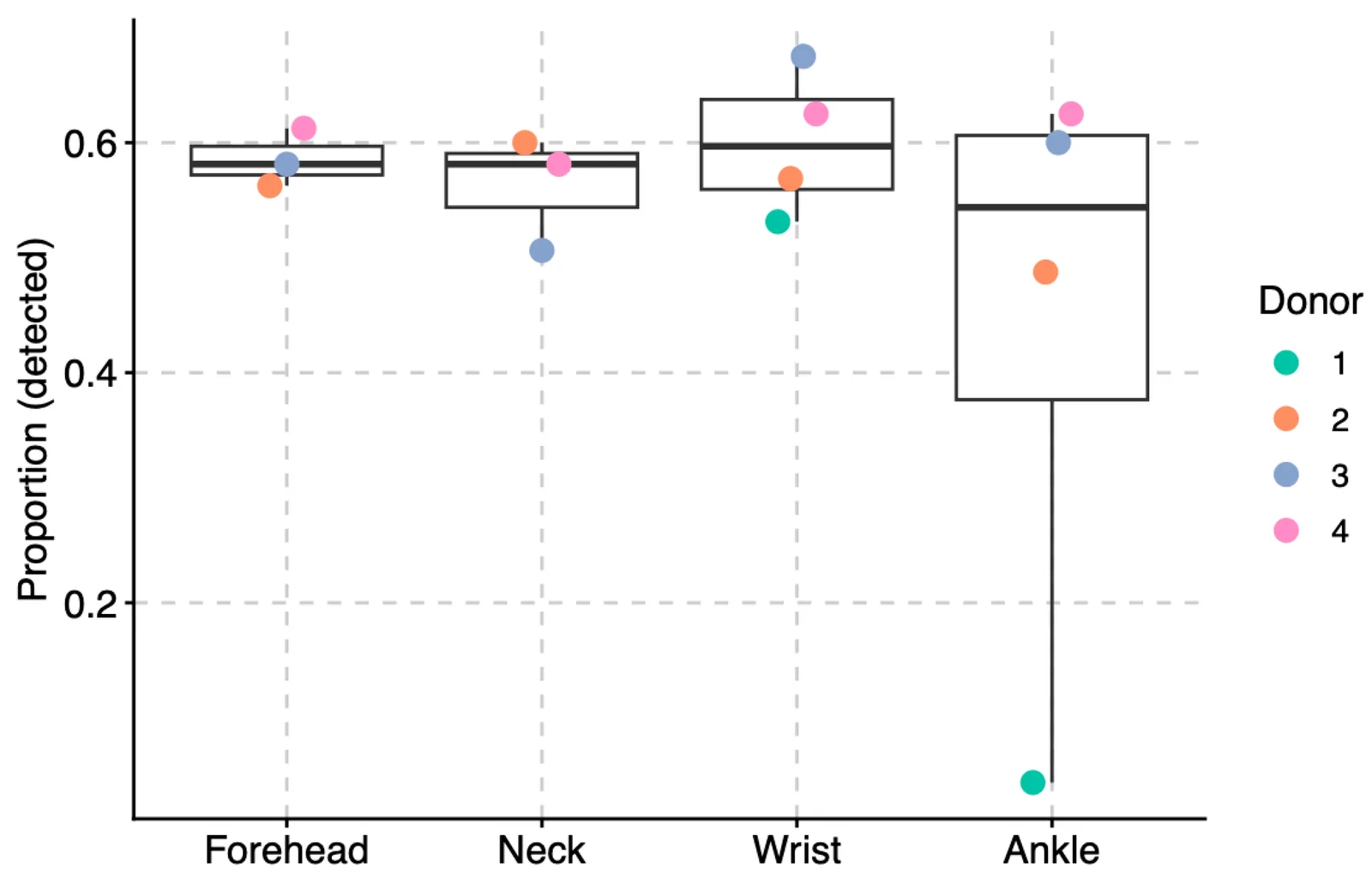
Boxplot showing the proportion of detected VOCs at each skin sampling site (forehead, neck, wrist, and ankle).

**Figure 4:**
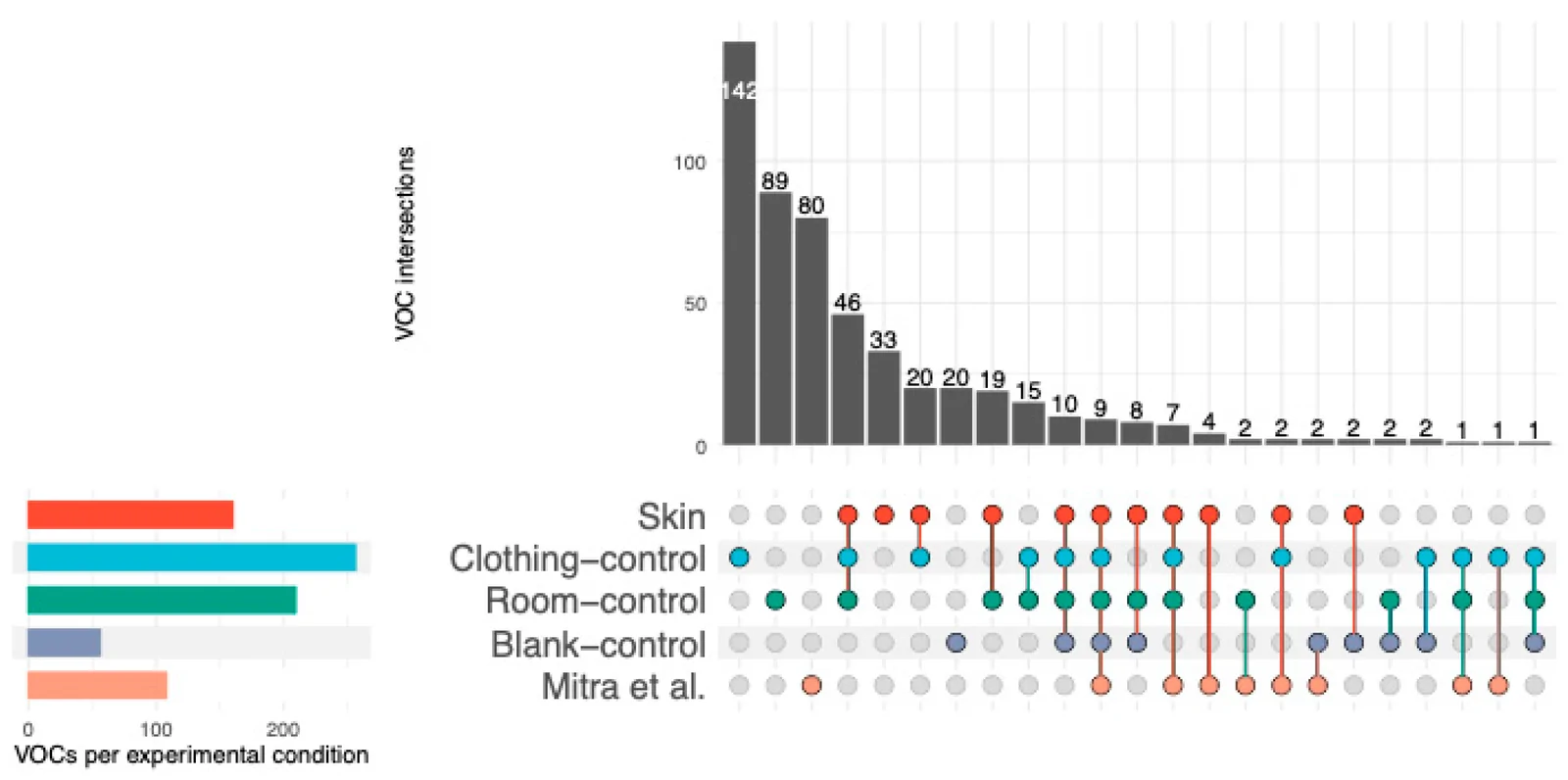
Upset plot of detected VOCs in PDMS and curated VOCs in the literature. Detected represents detected VOCs by skin PDMS, and other control settings, including blank control, room control, and clothing control. The compartment labeled as “Mitra et al.” represents curated VOCs in the literature [6].

## Data Availability

R scripts used for data preprocessing and analysis are available from https://github.com/Kamaleswaran-Lab/Skin_VOC_PDMS (accessed on 21 September 2026). The datasets underlying this article will be made available from the corresponding author upon reasonable request.

## Supplementary Materials

The following supporting information can be downloaded at https://www.mdpi.com/article/10.3390/metabo16100728/s1. Supplementary Methods. Supplementary Table S1. Putatively annotated VOCs detected across experimental conditions. Supplementary Table S2. Higher-confidence putatively annotated VOCs detected across experimental conditions. Supplementary Figure S1. Sensitivity analysis using compound confidence filter.
